# Cancer chemotherapy and beyond: Current status, drug candidates, associated risks and progress in targeted therapeutics

**DOI:** 10.1016/j.gendis.2022.02.007

**Published:** 2022-03-18

**Authors:** Uttpal Anand, Abhijit Dey, Arvind K. Singh Chandel, Rupa Sanyal, Amarnath Mishra, Devendra Kumar Pandey, Valentina De Falco, Arun Upadhyay, Ramesh Kandimalla, Anupama Chaudhary, Jaspreet Kaur Dhanjal, Saikat Dewanjee, Jayalakshmi Vallamkondu, José M. Pérez de la Lastra

**Affiliations:** a Department of Life Sciences, Ben-Gurion University of the Negev, Beer-Sheva 84105, Israel; b Department of Life Sciences, Presidency University, Kolkata, West Bengal 700073, India; c Center for Disease Biology and Integrative Medicine, Faculty of Medicine, The University of Tokyo, Tokyo 113-0033, Japan; d Department of Botany, Bhairab Ganguly College (affiliated to West Bengal State University), Kolkata, West Bengal 700056, India; e Faculty of Science and Technology, Amity Institute of Forensic Sciences, Amity University Uttar Pradesh, Noida 201313, India; f Department of Biotechnology, School of Bioengineering and Biosciences, Lovely Professional University, Phagwara, Punjab 144411, India; g Institute of Endocrinology and Experimental Oncology (IEOS), National Research Council (CNR), Department of Molecular Medicine and Medical Biotechnology (DMMBM), University of Naples Federico II, Naples 80131, Italy; h Department of Biochemistry, School of Life Sciences, Central University of Rajasthan, Bandar Sindari, Kishangarh Ajmer, Rajasthan 305817, India; i CSIR-Indian Institute of Chemical Technology, Hyderabad, Telangana 500007, India; j Department of Biochemistry, Kakatiya Medical College, Warangal, Telangana 506007, India; k Orinin-BioSystems, LE-52, Lotus Road 4, CHD City, Karnal, Haryana 132001, India; l Department of Computational Biology, Indraprastha Institute of Information Technology Delhi (IIIT-D), Okhla Industrial Estate, Phase III, New Delhi 110020, India; m Advanced Pharmacognosy Research Laboratory, Department of Pharmaceutical Technology, Jadavpur University, Kolkata 700032, India; n Department of Physics, National Institute of Technology-Warangal, Warangal, Telangana 506004, India; o Biotechnology of Macromolecules Research Group, Instituto de Productos Naturales y Agrobiología, IPNA-CSIC, San Cristóbal de La Laguna 38206, Tenerife, Spain

**Keywords:** Antimicrobial peptides, Cancer therapies, Clinical trials, Combination therapy, Immunotherapy, Patient survival, Personalized medicine, Targeted drug delivery

## Abstract

Cancer is an abnormal state of cells where they undergo uncontrolled proliferation and produce aggressive malignancies that causes millions of deaths every year. With the new understanding of the molecular mechanism(s) of disease progression, our knowledge about the disease is snowballing, leading to the evolution of many new therapeutic regimes and their successive trials. In the past few decades, various combinations of therapies have been proposed and are presently employed in the treatment of diverse cancers. Targeted drug therapy, immunotherapy, and personalized medicines are now largely being employed, which were not common a few years back. The field of cancer discoveries and therapeutics are evolving fast as cancer type-specific biomarkers are progressively being identified and several types of cancers are nowadays undergoing systematic therapies, extending patients' disease-free survival thereafter. Although growing evidence shows that a systematic and targeted approach could be the future of cancer medicine, chemotherapy remains a largely opted therapeutic option despite its known side effects on the patient's physical and psychological health. Chemotherapeutic agents/pharmaceuticals served a great purpose over the past few decades and have remained the frontline choice for advanced-stage malignancies where surgery and/or radiation therapy cannot be prescribed due to specific reasons. The present report succinctly reviews the existing and contemporary advancements in chemotherapy and assesses the status of the enrolled drugs/pharmaceuticals; it also comprehensively discusses the emerging role of specific/targeted therapeutic strategies that are presently being employed to achieve better clinical success/survival rate in cancer patients.

## Introduction

Cancer is a heterogeneous and multifactorial disease in which a series of genomic/molecular alterations cause the uncontrolled growth and proliferation of the cells, causing a rapid increase in tissue mass in the affected parts of the body. Under normal conditions, a cell gets signals to die and to supplant the organism with a young and healthier cell. Cancer cells grow using the body's oxygen and supplements, depriving other cells of regular supplements and growth factors. These cells can turn the microenvironment in their favor, deceive the immune system of the body, and can exploit the physiology of other cells to accommodate their needs.^1^, ^2^, ^3^, ^4^, ^5^, ^6^ Some of the biomarkers which are currently been used to detect cancer including human epididymis protein 4 (HE4), carcinoembryonic antigen (CEA), legumain, mesothelin, osteopontin, and vitamin E-binding plasma protein. A plethora of anti-cancer drugs and natural medicinal compounds have been devised over the years, which can suppress tumor growth through diverse mechanisms. Some of the drugs/compounds work on crucial cellular enzymes, while others may alter cell metabolism. They have also shown the potential to interfere with some critical cellular processes, e.g., programmed cell death/apoptosis, drug resistance, DNA damage, DNA replication, or immune reactions. These drugs have distinct modes of action and selectivity for multiple cancer types^7^, ^8^, ^9^, ^10^, ^11^, ^12^; however, subtle alteration to their chemical structure could abolish their anti-cancer potency.^13^^,^^14^

The idea of chemotherapy (utilizing toxic compounds and drugs to destroy cancerous cells) came into existence after the reports of mustard gas killing lymphatic tissues and bone marrow. The effects were later validated in mice using an effective derivative of the gas (nitrogen mustard) that showed effective regression of lymphoma tissues.^15^, ^16^, ^17^ The first patient receiving this nitrogen mustard was forty-eight years old lymphosarcoma patient whose cancer softened and cleared initially. Unfortunately, later he died after relapsing, but this secret military trial at Yale University paved the way for chemicals in treating cancers and developing the field of cancer chemotherapy for treating a variety of cancers.^18^, ^19^, ^20^, ^21^

Chemotherapy primarily works by circumventing the cancer cells from further growth and division. Cancer cells usually divide and grow at a much-accelerated rate than normal cells and are physiologically possess very high endogenous stress. Therefore, the drugs can destroy them rapidly and more effectively in comparison to other surrounding cells. Some of the promising inhibitor therapies which have been used to treat solid cancers are polyadenosine diphosphate-ribose polymerase inhibitors, angiogenesis inhibitors, histone deacetylase (HDAC) inhibitors, mechanistic target of rapamycin (mTOR) inhibitors, poly (adenosine di-phosphate-ribose) polymerase (PARP) inhibitors, p53/mouse double minute 2 homolog (MDM2) inhibitors, hedgehog pathway blockers, tyrosine kinase inhibitors and proteasome inhibitors.^22^^,^^23^ Chemotherapy may have different variations, which can affect the target cells in distinct manners.^24^ Some of the treatments may directly alter the quality of cellular proteins, rendering them non-functional and affecting major cellular physiological pathways. Major chaperone repressors, autophagy suppressors, or proteasome inhibitors belong to these classes of molecules.^25^, ^26^, ^27^, ^28^, ^29^, ^30^, ^31^ Other drugs may target some essential hormones and interfere with the body's overall metabolism.^32^^,^^33^ The 2018 Nobel Prize attributed to immune checkpoint therapy research has highlighted the importance of immunotherapy, which underlies the possibilities of modulating our immune system in our favor to cope up with the cancer-like conditions and fight back against the disease.^24^^,^^34^

The selection of chemo-preventive drugs or a combination of drugs is mostly based on the type and stage of cancer. The primary objective of these drugs is to neutralize the cancerous cells and ameliorate the stress caused by the tumor's growth. The dosage and timespan of the treatment are two important points of consideration. It has been observed that the medications are given at very high doses causing numerous side effects and harm to other healthy cells.^35^ One major challenge is the relapse of the disease. During reoccurrence, it has been widely observed that the cancer cells attain drug resistance following longer exposure to the drugs. Despite enormous applications, most chemotherapies are given to prolong and ease the patient's survival, hence called palliative chemotherapy.^21^ However, prolonged exposure to these drugs may have excessively adverse effects on a patient's physical and mental health, making it difficult to continue the ongoing treatment. Oncologists, for the most part, provide these medications with possible time intervals in between, which also gives non-transformed cells a chance to mend.^36^ In most cases, a team of specialists, including radiologists, nutritionists, psychologists, and the primary consultant collectively decide the treatment plans that include drug combinations, dosage, duration of cycles, and additional supplements.

**Figure 1:**
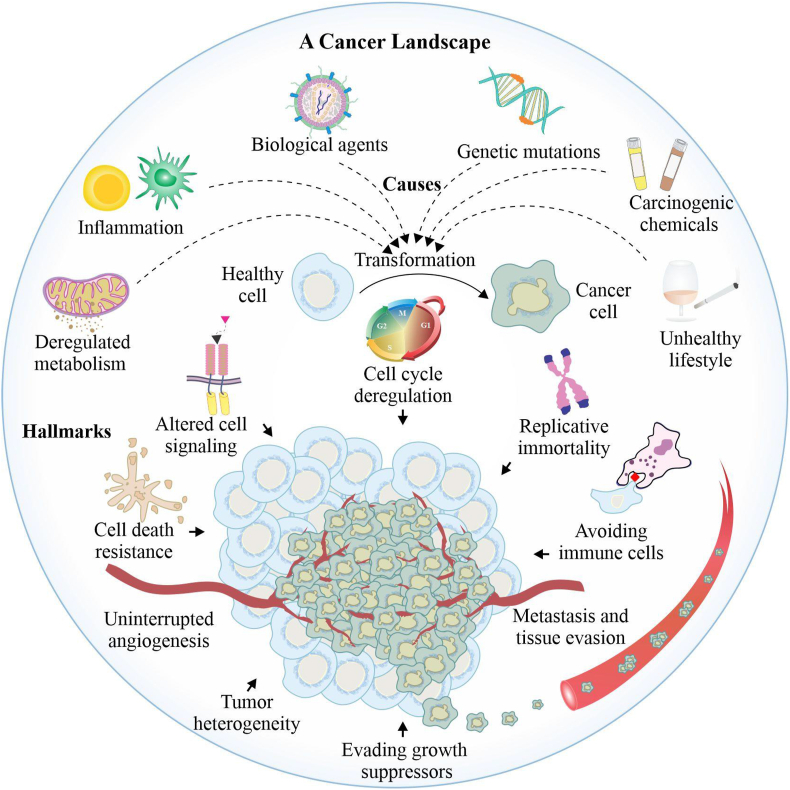
Schematic diagram showing different intrinsic and extrinsic biological/molecular events potentiating the transformation of a normal cell to the cancer cell, while the lower part of this diagram showing different hallmarks of a transformed cancer cell.

This report mainly reviews various aspects of cancer chemotherapy, including various applications of the drugs/chemotherapeutic agents that are already in practice. We also succinctly discussed various side effects and drawbacks of these treatment plans and further provided an overview of recent advancements in interventional strategies by focusing on targeted drug therapy, immunotherapy, personalized and integrative medicines. Figure 1 depicts the significant intrinsic and extrinsic cellular and molecular events causing the transformation of a normal cell to a cancer cell and its distinct hallmarks.

## Cancer chemotherapeutics: what is known so far?

The idea of cancer as a disease was known to Hippocrates, the father of medicine, around 400 BC. He later coined the term ‘carcinos’ due to the similarity of cancer tissue growths with crabs. The term was later replaced with the Latin word cancer by Celsus. Many ancient pieces of literature have described several kinds of malignancies in humans, and multiple treatment approaches were also in use in different parts of the ancient world, as found in the literature of that era.^37^^,^^38^ However, the progress of strategies to treat the disease was more or less static until the beginning of the twentieth century.^39^ The extract of the plant *Colchicum autumnale* was used by Greek physicians to dissolve the tumor mass. Interestingly, in the 1930s, the active compound in the extract, colchicine, was reported that can interfere with microtubule assembly and may work as a potential drug.^40^ Vincristine and vinblastine are other similar compounds that were medicinally recognized as possible anti-tumor agents a long time ago.^41^ The ancient pieces of the literature suggest that the Greek and Roman physicians have contributed enormously to the identification and characterization of disease. Arabic, Indian, and Chinese medicinal archives have also paved the way for multiple medieval and modern approaches to understand and treat the disease. Interestingly, for centuries, most of the approaches remain limited to cauterization, bloodletting, surgery, herbal medicine, etc.^42^ In modern onco-medicine, which was evolved during the Second World War, many active compounds were reported following the detailed characterization of the disease at the genetic and molecular level. The initial decades have seen enormous debate on the use of cytotoxic agents as drug candidates; however, advancements were made, and success came in the form of Eli Lily introducing *Vinca* alkaloids in the 1960s, which was then followed by procarbazine.^43^ In the following years, the idea of combination therapy was introduced for acute lymphocytic leukemia and non-Hodgkin's lymphoma patients that were also supplemented with supportive medical care to enhance the quality of life.^21^

**Figure 2:**
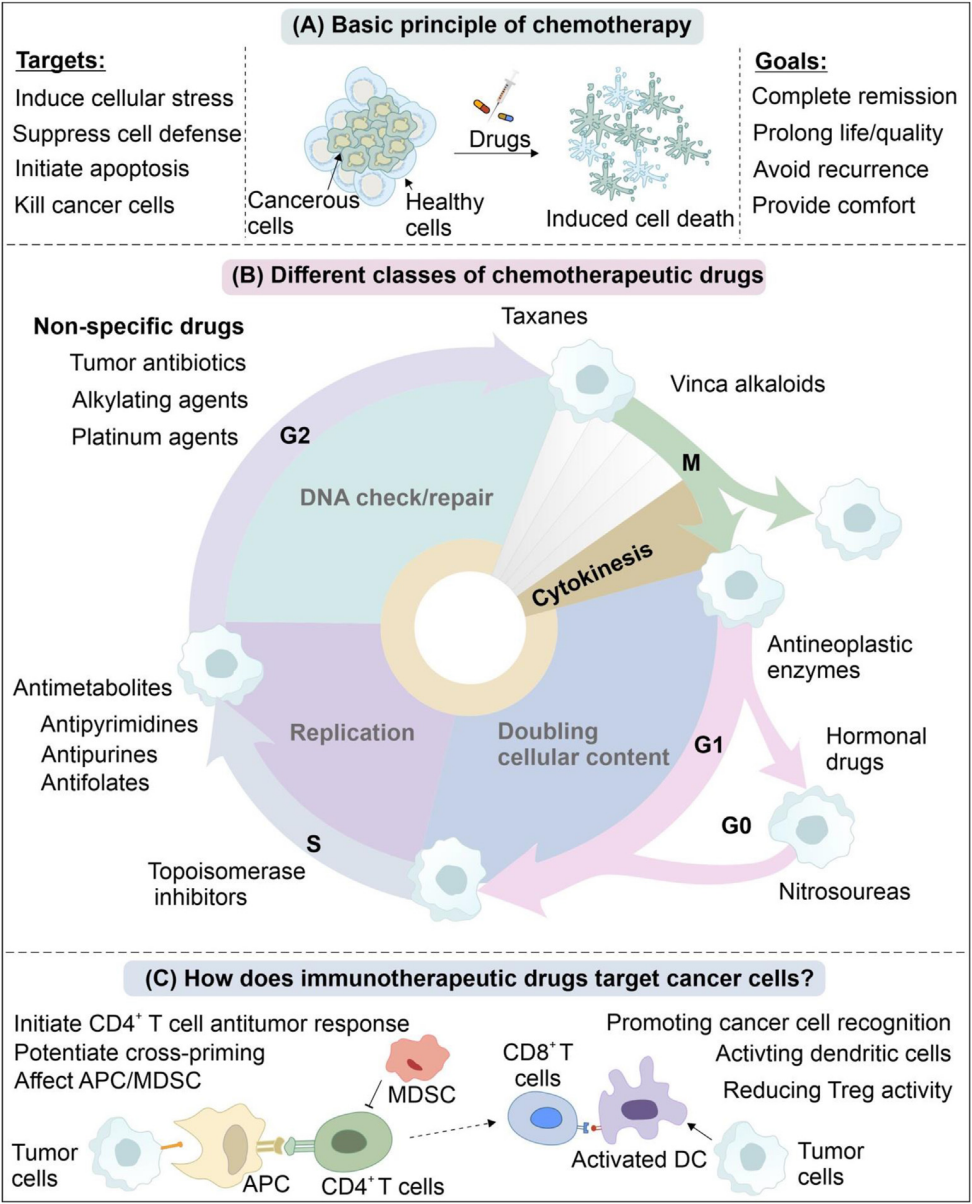
Schematic diagram showing the basic principles of chemotherapy **(A)**, different classes of chemotherapeutic agents/drugs **(B)**, and immunotherapeutic mechanism(s) of targeting cancer cells **(C)**.

In the second half of the twentieth century, a series of reports have identified numerous mutations, external stimuli (toxic chemicals, viruses, etc.), metabolic alterations, etc. as the underlying causes of cancer.^44^^,^^45^ Identification of a plethora of cellular pathways that are affected in various cancer types has given different molecular alternatives to target and develop effective drugs (Fig. 2).^46^, ^47^, ^48^ Several modes of medications have also been introduced and tried for drug administration based on the types and stages of the diagnosed cancer. Here, we discuss the available drug medications, following which we have revisited the chemotherapeutic agents, which have substantially pushed the field forward and extended the lives of millions in the past several decades.

Intravenous (IV) chemotherapy requires drug infusion straightforwardly into the patient's veins that may take from minutes to a few hours. Some medications can also be given either as pills or fluid regularly or at some intervals.^49^ Intramuscular shots and intra-abdominal administration are other methods of giving chemotherapeutic medications to target the specific locations of tumor mass.^50^ In addition, a new technology called pressurized intraperitoneal aerosol chemotherapy has been developed to efficiently deliver intraperitoneal chemotherapy for end-stage peritoneal metastases patients.^51^ Topical treatment methods imply providing drugs through the skin surface. The anti-cancer compounds derived from either natural sources or their synthetic chemical analogs can kill cancer cells or stop their development.^52^, ^53^, ^54^ Notably, various other approaches are emerging to treat the cancer cells, including small molecule inhibitors,^55^ a combination of anti-cancer agents and immunotherapy,^56^ nanotechnology-based drugs,^57^, ^58^, ^59^ and organo-seleno compounds (Fig. 2).^31^

Nevertheless, the selectivity of most drug particles is restricted, and such medications are considered one of the most harmful medications utilized in the treatment.^60^^,^^61^ Since the initial findings of modern anticancer drug discoveries in the 1940s, continuous efforts are being made to treat this life-threatening disease. Advances in scientific methods and techniques have brought new treatment and diagnostic tools and advanced patient care measures (Fig. 2).^62^, ^63^, ^64^, ^65^, ^66^, ^67^, ^68^, ^69^ Treatment plans may have palliation as one strategy that may lead to shrinkage of the obvious tumor, easing side effects, and increasing the life quality of patients. Figure 2 describes various ways of providing anti-cancer medications to patients depending upon their type and stage of the disease.

Along with primary treatment, adjuvant therapy is also given to the patients for additionally combating the disease through supportive strategies.^70^^,^^71^ Surgery and radiation can be given to patients to supplement a chemotherapeutic plan to curb the disease by destroying shrouded cancer cells that could not be targeted otherwise.^72^ Sometimes adjuvant chemotherapy is given to patients after surgery or radiation or both to lessen the chances of recurrence.^73^, ^74^, ^75^ Adjuvant chemotherapy starts typically within three to five weeks of the surgical removal of a tumor and has distinctive treatment lengths relying upon the disease. Although chemotherapy has significantly improved overall survival, patients experience a wide range of physical and psychological symptoms that impact their quality of life. Symptoms seldom occur in isolation. Chemotherapy-induced hair loss remains greatly feared, with a negative impact on the well-being of many cancer patients. Cancer- and chemotherapy-induced poor appetite is usually the result of taste changes, mouth sores, nausea and vomiting, increased satiety, medication side effects, pain, fatigue, depressed mood, and anxiety.

## Basic principles of chemotherapy: years of research, miles to go

The cancer cell metabolic mechanisms functionally overlap with the host cells, so cancer treatment is very challenging.^76^^,^^77^ Designing drugs or therapeutics mainly focuses on selectivity, which can specifically kill the cancerous cells without affecting the non-cancerous cells (Fig. 2). In a way, the anti-cancer therapy may resemble antimicrobials, but the cancerous cells and the bacterial cells have differences in terms of the metabolic and physiological characters.^78^, ^79^, ^80^ For our immune cells, bacterial cell recognition is straightforward, while our transformed cells can hide cell surface receptors to evade the immune system.^81^ Early-stage detection of cancers increases the chances of treatment and survival. Therefore, recognizing and diagnosing those transformed cells remains one of the most challenging tasks in oncological research. At early stages, most known biomarkers remain undetectable, and cancer cells' ability to hide from immune cells freezes our immune responses.^82^^,^^83^

In a tumor subpopulation, transformed cells differ drastically from the other cells in terms of physiology, metabolism, proliferation rate, etc.^84^, ^85^, ^86^ Besides that, the genetic diversity may pose additional challenges to target all kinds of cells in a particular tissue mass owing to the tumor heterogeneity.^87^^,^^88^ A combination of drug therapy is applied to address this challenge, and the regime is devised according to the cancer phase and type (Fig. 2).^89^^,^^90^

In the subsequent section, we provide a comprehensive overview of various chemotherapeutic agents which have served the purpose for several decades in different cancer types. These drugs could be classified in different ways based on their chemical nature/source, molecular target, mechanism/mode of action, or effectiveness in model systems against various malignancies/cancer types (Table 1).

**Table 1:** Table listing chemotherapeutic drugs/agents used in current clinical practice along with details of their molecular targets, mode of action, and investigated model systems across diverse malignancies.

Drugs/Compound	Molecular targets	Mechanism of action	Studied model system	Reference/Source
Antineoplastic alkylating agents				
Altretamine	DNA polymerase alpha catalytic subunit Ribonucleoside-diphosphate reductase large subunit DNA	Inhibitor Inhibitor Other/unknow	B-cell chronic lymphocytic, leukemia/prolymphocytic leukemia/small lymphocytic lymphoma refractory, and refractory non-Hodgkin's lymphoma	^278^
Bendamustine	DNA	Intra- and inter-strand crosslinking	Chronic lymphocytic leukaemia (CLL), follicular non-Hodgkin's lymphoma refractory, refractory Hodgkin lymphoma, refractory Mantle cell lymphoma, Waldenström's macroglobulinemia (WM), recurrent multiple myeloma, and refractory indolent B cell non-Hodgkin lymphoma	^279^
Busulfan	DNA	Cross-linking/alkylation	Chronic myelogenous leukemia	^279^
Carboplatin	DNA	Cross-linking/alkylation	Advanced cervical cancer, advanced Endometrial Cancer, advanced esophageal cancers, advanced head and neck Cancer, advanced melanoma, advanced non-small cell lung cancer, advanced ovarian carcinoma, advanced Sarcoma, metastatic breast cancer, neuroendocrine carcinoma of the skin, pleural mesothelioma, refractory Hodgkin lymphoma, retinoblastoma, advanced bladder cancer, advanced small cell lung cancer, advanced testicular cancer, advanced thymoma, advanced thymic carcinoma, and refractory non-Hodgkin's lymphoma	^279^
Carmustine	DNA Glutathione reductase, mitochondrial RNA	Other/unknown inhibitor Other/unknown	Glioblastoma, brainstem glioma, medulloblastoma, astrocytoma, ependymoma and metastatic brain tumors, multiple myeloma, Hodgkin's disease, non-Hodgkin's lymphomas, and may be used on the skin (topically) for cutaneous T-cell lymphoma	^280^
Chlorambucil	DNA	Cross-linking/alkylation	Chronic lymphocytic leukemia, lymphomas, and Hodgkin's disease	^279^
Cisplatin	DNA DNA-3-methyladenine glycosylase Alpha-2-macroglobulin Serotransferrin Copper transport protein ATOX1	Cross-linking/alkylation Not Available Not Available Not Available Not Available	Testicular cancer, ovarian cancer, bladder cancer, head and neck cancer, esophagus cancer, small cell and non-small cell lung cancer, non-Hodgkin's lymphoma and trophoblastic neoplasms	^279^
Cyclophosphamide	DNA Nuclear receptor subfamily 1 group I member 2	Cross-linking/alkylation Not Available	Lymphoma, multiple myeloma, leukemia, ovarian cancer, breast cancer, small cell lung cancer, neuroblastoma, and sarcoma.	^279^
Dacarbazine	DNA DNA polymerase alpha subunit B 6-phosphogluconate dehydrogenase, decarboxylating	Cross-linking/alkylation Other/unknown Inhibitor	Melanoma skin cancer, soft tissue sarcoma, Hodgkin lymphoma	^279^
Daunorubicin	DNA DNA topoisomerase 2-alpha DNA topoisomerase 2-beta	Intercalation Inhibitor Inhibitor	Acute nonlymphocytic leukemia in adults and acute lymphocytic leukemia in adults and children	^279^
Decitabine	DNA DNA (cytosine-5)-methyltransferase 1 DNA (cytosine-5)-methyltransferase 3A DNA (cytosine-5)-methyltransferase 3B	Other/unknown Inhibitor Inhibitor Inhibitor	Myelodysplastic syndromes, and acute myeloid leukemia (AML)	^279^^,^^281^
Doxorubicin	DNA DNA topoisomerase 2-alpha Nucleolar and coiled-body phosphoprotein 1	Intercalation Inhibitor Not available		^279^
Epirubicin	DNA topoisomerase 2-alpha DNA	Inhibitor Intercalation	Breast cancer	^279^^,^^282^
Etoposide	DNA topoisomerase 2-alpha DNA topoisomerase 2-beta	inhibitor Inhibitor	Testicular cancer, lung cancer, lymphoma, nonlymphocytic leukemia, neuroblastoma, and ovarian cancer, Kaposi's sarcoma, Ewing's sarcoma and glioblastoma multiforme	^279^
Idarubicin	DNA DNA topoisomerase 2-alpha	Intercalation Inhibitor	Acute myelogenous, acute lymphoblastic and chronic myelogenous	^279^
Ifosfamide	DNA Nuclear receptor subfamily 1 group I member 2	Other/unknown Not available	Germ cell testicular cancer, soft tissue sarcoma, acute lymphocytic leukemia, bladder cancer, bone cancer, pancreatic cancer, stomach cancer, breast cancer, cervical cancer, endometrial cancer, lung cancer, ovarian cancer, neuroblastoma, lymphoma, and Wilms' tumor	^279^
Irinotecan	DNA topoisomerase 1, DNA topoisomerase I, mitochondrial	Inhibitor Inhibitor	Metastatic colorectal cancer, other colorectal cancers and non-small/small lung cancers	^279^
Lomustine	DNA Stathmin-4	Cross-linking/alkylation Antagonist	Metastatic brain tumors, Hodgkin's and non-Hodgkin's lymphoma	^279^
Mechlorethamine	DNA	Intercalation	Hodgkin's disease, chronic lymphocytic leukemia, chronic myelogenous leukemia, small cell lung cancer, medulloblastoma, and non-Hodgkin's lymphoma	^283^
Melphalan	DNA	Cross-linking/alkylation	Multiple myeloma, ovarian cancer, breast cancer, melanoma, and amyloidosis	^284^
Mitomycin C	DNA	Cross-linking/alkylation	Gastric cancer, anal and colon cancer, breast cancer, non-small cell lung cancer, head and neck cancer, small bladder papillomas, pancreatic cancer, cervical cancer	^279^
Mitoxantrone	DNA DNA topoisomerase 2-alpha	Intercalation Inhibitor	Advanced, hormone-refractory prostate cancer, acute nonlymphocytic leukemia (ANLL), breast cancer and non-Hodgkin's lymphoma.	^285^
Oxaliplatin	DNA	Cross-linking/alkylation	Metastatic or recurrent colorectal cancer, metastatic pancreatic cancer and metastatic gastric cancer.	^286^
Temozolomide	DNA	Cross-linking/alkylation	Brain tumors (astrocytomas) in adult patients, metastatic melanoma and glioblastoma multiforme	^287^
Topotecan	DNA topoisomerase 1 DNA topoisomerase I, mitochondrial DNA	Inhibitor Inhibitor Intercalation	Ovarian cancer, small cell lung cancer, and certain types of cervical cancer	^288^
Thiotepa	DNA	Cross-linking/alkylation	Adenocarcinoma of the breast, adenocarcinoma of the ovary, papillary thyroid cancer and bladder cancer, Hodgkin's and non-Hodgkin's lymphomas	^289^
Trabectedin	DNA	Binder	Soft tissue sarcoma, and ovarian cancer	^290^
Antineoplastic antimetabolites				
Azathioprine	Ras-related C3 botulinum toxin substrate 1	Modulator	Leukemia and lymphomas	^291^
Cladribine	Ribonucleoside-diphosphate reductase large subunit Ribonucleoside-diphosphate reductase subunit M2 Ribonucleoside-diphosphate reductase subunit M2 B DNA DNA polymerase alpha catalytic subunit DNA polymerase epsilon catalytic subunit A DNA polymerase epsilon subunit 2 DNA polymerase epsilon subunit 3 DNA polymerase epsilon subunit 4 Purine nucleoside phosphorylase	Inhibitor Inhibitor Inhibitor Other/unknown Inhibitor Inhibitor Inhibitor Inhibitor Inhibitor Inducer	Drug-resistant T-cell prolymphocytic leukemia	^292^
Clofarabine	DNA polymerase alpha catalytic subunit Ribonucleoside-diphosphate reductase large subunit DNA	Inhibitor Inhibitor Other/unknown	Relapsed or refractory acute lymphoblastic leukaemia, acute myeloid leukaemia, juvenile myelomonocytic leukaemia, and myelodysplastic syndrome	^293^
Fludarabine	Ribonucleoside-diphosphate reductase large subunit DNA polymerase alpha catalytic subunit DNA Deoxycytidine kinase	Inhibitor Inhibitor Incorporation into and destabilization Agonist	Chronic lymphocytic leukemia, non-Hodgkin's lymphoma, acute myeloid leukemia, and acute lymphocytic leukemia	^294^
Gemcitabine	DNA Ribonucleoside-diphosphate reductase large subunit Thymidylate synthase UMP-CMP kinase	Cross-linking/alkylation Inhibitor Inhibitor Inhibitor	Breast cancer, ovarian cancer, non-small cell lung cancer, pancreatic cancer and bladder cancer	^295^
Mercaptopurine	Hypoxanthine-guanine phosphoribosyltransferase Amidophosphoribosyltransferase Inosine-5′-monophosphate dehydrogenase	Inhibitor Inhibitor Inhibitor	Acute lymphocytic leukemia (ALL), chronic myeloid leukemia (CML), Crohn's disease and ulcerative colitis.	^296^
Nelarabine	DNA DNA polymerase alpha catalytic subunit	Incorporation into and destabilization Inhibitor	Acute T-cell lymphoblastic leukemia	^297^
Pentostatin	Adenosine deaminase	inhibitor	Hairy cell leukaemia refractory to alpha interferon	^298^
Tioguanine	DNA	Intercalation	Acute leukemia	^299^
Pyrimidine-derived antimetabolites (pyrimidine antagonists)				
Capecitabine	Thymidylate synthase DNA RNA	Inhibitor Incorporation into and destabilization Incorporation into and destabilization	Metastatic breast, gastric and colorectal cancers	^99^
Cytarabine	DNA polymerase beta DNA	Inhibitor Cross-linking/alkylation	Acute myeloid leukemia (AML), acute lymphocytic leukemia (ALL), chronic myelogenous leukemia (CML) and non-Hodgkin's lymphoma	^97^
Decitabine	DNA DNA (cytosine-5)-methyltransferase 1 DNA (cytosine-5)-methyltransferase 3A DNA (cytosine-5)-methyltransferase 3B	Other/unknown Inhibitor Inhibitor Inhibitor	Myelodysplastic syndromes (MDS)	^101^
Gemcitabine	DNA Ribonucleoside-diphosphate reductase large subunit Thymidylate synthase UMP-CMP kinase	Cross-linking/alkylation Inhibitor Inhibitor Inhibitor	Metastatic ovarian cancer, metastatic non-small cell lung cancer, and metastatic pancreatic adenocarcinoma	^100^
Fluorouracil	Thymidylate synthase DNA RNA	Other/unknown Incorporation into and destabilization Incorporation into and destabilization	Actinic keratosis (AK), breast cancer, malignant neoplasm of colon, malignant neoplasm of pancreas, malignant neoplasm of stomach, rectal carcinoma, superficial basal cell carcinoma, and hyperkeratotic actinic keratosis	^98^
Raltitrexed	Thymidylate synthase Folylpolyglutamate synthase, mitochondrial	Inhibitor Antagonist	Advanced Colorectal Cancer, Pleural Mesotheliomas	^102^
Tegafur	Thymidylate synthase	Inhibitor	Advanced Gastric Cancer	^103^
Other antineoplastic agents (belong to one or multiple drug classes)				
Arsenic trioxide (Trisenox®)	Inhibitor of nuclear factor Kappa-B kinase subunit beta Thioredoxin reductase 1, cytoplasmic Transcription factor AP-1 G1/S-specific cyclin-D1 Mitogen-activated protein kinase 3 Mitogen-activated protein kinase 1 inducer RAC-alpha serine/threonine-protein kinase Cyclin-dependent kinase inhibitor 1 Histone deacetylase 1 Protein PML	Inducer Inhibitor Inducer Antagonist Inducer Inducer Inducer Not available Not available Not available	Refractory Acute Promyelocytic Leukemia	^300^
Hydroxyurea	Ribonucleoside-diphosphate reductase large subunit	Inhibitor	Melanoma, resistant chronic myelocytic leukemia, and recurrent, metastatic, or inoperable carcinoma of the ovary and Sickle-cell anemia	^301^
Paclitaxel (Taxol®)	Tubulin beta-1 chain Apoptosis regulator Bcl-2 Microtubule-associated protein 4 Microtubule-associated protein 2 Microtubule-associated protein tau Nuclear receptor subfamily 1 group I member 2	Inhibitor Inhibitor Not Available Not Available Not Available Inducer	Kaposi's sarcoma and cancer of the lung, ovarian, and breast	^302^
Docetaxel (Taxotere®)	Tubulin beta-1 chain Apoptosis regulator Bcl-2 Microtubule-associated protein 2 Microtubule-associated protein 4 Microtubule-associated protein tau Nuclear receptor subfamily 1 group I member 2	Not available Not available Not available Not available Not available Binder	Locally advanced or metastatic breast cancer, locally advanced or metastatic non-small cell lung cancer, hormone refractory metastatic prostate cancer, gastric adenocarcinoma and head and neck cancer	^303^
Ixabepilone (Ixempra®)	Tubulin beta-3 chain	Inhibitor	Breast cancer, head and neck cancer, melanoma, lung cancer, non-Hodgkin's lymphoma, prostate cancer, renal cell carcinoma	^279^
Methotrexate	Dihydrofolate reductase Humans Thymidylate synthase Bifunctional purine biosynthesis protein PURH	Inhibitor Inhibitor Inhibitor	Pediatric acute lymphoblastic leukemia, gestational choriocarcinoma, chorioadenoma destruens, breast cancer, epidermoid cancer of the head and neck, lung cancer, and advanced non-Hodgkin's lymphoma	^304^
Pemetrexed (Alimta®)	Thymidylate synthase Bifunctional purine biosynthesis protein PURH Dihydrofolate reductase Trifunctional purine biosynthetic protein adenosine-3	Inhibitor Inhibitor Inhibitor Inhibitor	Malignant pleural mesothelioma, and metastatic non-small cell lung cancer (NSCLC)	^305^
Streptozocin	DNA Solute carrier family 2, facilitated glucose transporter member 2 O-GlcNAcase BT_4395 Bifunctional protein NCOAT	Cross-linking/alkylation Ligand Antagonist Not available	Malignant neoplasms of pancreas	^306^
Vinblastine	Tubulin alpha-1A chain Tubulin beta chain Tubulin delta chain Tubulin gamma-1 chain Tubulin epsilon Transcription factor AP-1	Adduct Adduct Adduct Adduct Adduct Other/unknown	Breast cancer, testicular cancer, lymphomas, neuroblastoma, Hodgkin's and non-Hodgkin's lymphomas and Kaposi's sarcoma	^307^
Vincristine	Tubulin beta-1 chain	Inhibitor	Acute lymphocytic leukemia (ALL), Hodgkin lymphoma, non-Hodgkin's lymphomas, Wilms' tumor, neuroblastoma, rhabdomyosarcoma, relapsed Philadelphia chromosome-negative (Ph-) acute lymphoblastic leukemia (ALL)	^308^
Vindesine	Tubulin beta-1 chain	Inhibitor	Acute leukaemia, malignant lymphoma, Hodgkin's disease	^309^
Vinorelbine	Tubulin beta chain	Antagonist inhibitor	Advanced non-small cell lung cancer (NSCLC), relapsed or refractory Hodgkin lymphoma, metastatic squamous cell head and neck cancer, recurrent ovarian cancer, metastatic breast cancer	^310^

## Alkylating agents

One major class of the current drugs belongs to alkylating agents, which can interfere with the formation/linkage of DNA double strands.^91^ These drugs can cause it by transferring one alkyl group to the guanidine base in DNA (Fig. 2). Cross-linking of nucleic acids with proteins or peptides can likewise affect the DNA geometry, cause erroneous base pairing and DNA strand breakage, and eventually preventing the cell division and causing irreversible senescence.^92^ Mechanistically, alkylating agents in their electrophilic forms interact with cellular DNA and form covalent adducts, underlying their broader utility. Therefore, alkylating drugs are the frontline chemotherapeutic agents given against most of the cancer types, yet the greatest therapeutic value of their inhibition is known for slow-growing cancers. In Table 1, we described the common chemotherapeutic alkylating agents including the altretamine, chlorambucil, platinum drugs (cisplatin, carboplatin, oxaliplatin), doxorubicin, epirubicin, etoposide, mitoxantrone, cyclophosphamide, thiotepa, and busulfan that are in clinical practice and also provided details of their molecular targets, mode of action, and studied model systems in various malignancies/cancer-types.

## Antimetabolites

Antimetabolites are the next class of substances that may compete, replace, or inhibit some specific metabolites inside the cells and thus meddle with the cellular metabolism (Fig. 2).^93^ These molecules mostly possess a structure similar to a cellular metabolite or enzyme–substrate, usually identified and processed by an enzyme to meet the cellular needs.^94^ Tetrahydrofolates are formed from unreduced dietary folates by the enzyme dihydrofolate reductase. For example, various medications, such as aminopterin, methotrexate (amethopterin), pyrimethamine, trimethoprim, and triamterene, act directly on the production of these metabolites, hindering the enzyme from producing folate insufficiency.^95^ Methotrexate, an antifolate drug, is one of the exceptionally successful anti-cancer medications that represses dihydrofolate reductase (DHFR) and obstructs the transformation of dihydrofolic acid (DHFA) into tetrahydrofolic acid (THFA).^96^ This coenzyme activity is indispensable to the cells as they are essential for the nucleotide synthesis pathways. Methotrexate is an anti-inflammatory molecule and is hence used as a medication for many other inflammatory diseases, like rheumatoid arthritis and extreme psoriasis, apart from various cancer types. A detailed description of common antimetabolites is comprehensively provided in Table 1.

## Pyrimidine-derived antimetabolites (pyrimidine antagonists) and *Vinca* alkaloids

Pyrimidine-derived antimetabolites are a group of chemotherapeutic agents that interrupt DNA synthesis and exert their cytotoxicity to cancer cells (Fig. 2). For instance, cytarabine also known as cytosine arabinoside or Ara C interrupts DNA as well as RNA synthesis by displacing the cytosine with itself (that differs merely by its base sugar arabinose). Cytarabine/cytosine arabinoside is enrolled frequently in clinics to treat leukemia or non-Hodgkin's lymphoma.^97^ Some other notably pyrimidine-derived antimetabolites include fluorouracil, 5-fluorouracil (5-FU), capecitabine, floxuridine, gemcitabine, decitabine, raltitrexed, and tegafur (Table 1).^98^, ^99^, ^100^, ^101^, ^102^, ^103^ Being as pyrimidine or purine analogs having altered chemical groups, these drugs/compounds induce apoptosis while progressing through the S phase of the cell cycle by their misincorporation to RNA and DNA or by inhibiting the key enzymes required for nucleic acid synthesis. Among these enzymes, DNA polymerases, ribonucleotide reductase, and thymidylate synthetase are the main intracellular targets of the pyrimidine antimetabolites. 5-FU has been a promising pyrimidine analog that largely interrupts DNA and RNA synthesis. 5-FU that dependents on its incorporation to DNA inhibits mitosis and stimulates cell death in the dividing cells.

*Vinca* alkaloids, primarily extracted from several species under the *Vinca* genus, are mainly cell division inhibitors, which can interact with microtubular protein tubulin to obstruct its polymerization and meddle with the cytoskeleton, leading to stalling of mitotic division.^104^ The chromosomes do not separate efficiently during cell division, leading to metaphase capture. Vincristine and vinblastine are primary examples of this drug class with different degrees and ranges of anti-tumor activity (Table 1).^105^^,^^106^ Cancers treated with these drugs include acute leukemia, Hodgkin's and non-Hodgkin's lymphoma, rhabdomyosarcoma, Ewing's sarcoma, neuroblastoma, Wilms' tumor, miscellaneous myeloma, chronic leukemia, thyroid cancer, brain tumors, and several blood-related disorders.^107^

## Other antineoplastic drugs/agents

Besides these three established classes, several other antineoplastic agents are also enrolled in the clinical practice of cancer chemotherapy (Table 1). Given their clinical promises, in the subsequent section, we described the biological activities, targets, and mechanisms of key chemotherapeutic drugs that may belong to one or multiple drug classes and can affect more than one cellular pathway in the cancer cells and induce apoptosis by different mechanisms (Fig. 2). A succinct review of well-known anti-cancer drugs that are in routine clinical practice is provided as follows.

## Paclitaxel/Taxol

It is a complex diterpene taxane ring-containing drug acquired from the bark of the Western yew tree (*Taxus brevifolia*), which induces cytotoxic activity by a novel system.^108^ It increases the polymerization of tubulin, thereby inducing a mechanism inverse to that of the aforementioned alkaloids.^109^ It forestalls the microtubule's structural adjustment and depolymerization, bringing an inherent cytotoxic activity to the paclitaxel due to the significance of tubulin-microtubule dynamics.^110^ The confirmed antagonistic impacts of paclitaxel are reported on the metastatic ovarian and bosom carcinomas after the disappointment from first-line chemotherapy and backslide cases.^111^ It has also shown efficacy against the advanced stage tumors of the head and neck disease, small cell lung cancer, oesophageal adenocarcinoma, and prostate cancer (Table 1).^112^^,^^113^ Docetaxel is a more potent relative of paclitaxel with a comparable activity. It has been shown to provide benefits against the bosom and ovarian cancers, which get resistant to first-line chemotherapeutic drugs.^114^ Unfortunately, many side effects have been reported from the long exposure to these drugs, which mainly include neutropenia, arrhythmia, neuropathy, and cardiovascular breakdown (Table 2).^115^^,^^116^

**Table 2:** Table listing the adverse effects of various chemotherapy drugs, affected organs, their reported toxicities/side effects and current stage of their clinical progresses.

Drugs/Compound	Affected organ	Reported toxicity/side effects (frequency in >30% cases)	Current status	Reference/Sources
Alkylating agents/drugs				
Altretamine	Blood	Nausea and vomiting, Low blood counts, Peripheral neuropathy	Approved	^278^
Bendamustine	Blood	Low blood counts, Increase in bilirubin levels	Approved, Investigational	^311^^,^^312^
Busulfan	Blood	Low blood counts, Nausea and vomiting (usually mild with standard doses), Loss of fertility, Diarrhea (usually mild with standard doses), Poor appetite, Mouth sores	Approved, Investigational	^313^^,^^314^
Carboplatin	Cervix, skin, endometrium, esophagus, head and neck, lung, bladder, thymus, mesothelium, testicle	Low blood counts, Nausea and vomiting, Taste changes, Hair loss, Weakness, Blood test abnormalities, abnormal magnesium level	Approved	^315^
Carmustine	Brain, skin	Nausea and vomiting, Facial flushing, Pain and burning at the injection site, Low blood counts	Approved, Investigational	^280^
Chlorambucil	Blood	Low blood counts	Approved	^316^^,^^317^
Cisplatin	Testicle, ovary, head-neck, lung, blood, esophagus, trophoblast	Nausea and vomiting, Low blood counts, Kidney toxicity, Ototoxicity, Blood test abnormalities (low magnesium, low calcium, low potassium)	Approved	^318^^,^^319^
Cyclophosphamide	Blood, ovarian, breast, lung, brain	Low blood counts, Hair loss, Nausea and vomiting, Poor appetite, Discoloration of the skin or nails	Approved, Investigational	^320^
Dacarbazine	Skin, blood	Local pain, burning sensation and irritation at the needle site during the infusion, Low blood counts, Nausea and vomiting, Poor appetite, Elevation of blood liver enzymes	Approved, Investigational	^321^^,^^322^
Daunorubicin	Blood	Pain along the site where the medication was given, Urine may appear red, red-brown, orange or pink from the color of the medication for one to two days after you receive a dose, Low blood counts, Nausea or vomiting, Hair loss on the scalp or elsewhere on the body	Approved	^323^^,^^324^
Decitabine	Blood	Low blood counts, Fatigue, Fever, Nausea, Cough, Petechiae, Diarrhea, Constipation, Hyperglycemia	Approved, Investigational	^325^
Doxorubicin	Blood, breast, ovarian, bladder, stomach, bronchus	Pain along the site where the medication was given, Nausea or vomiting, Low blood counts, Hair loss on the scalp or elsewhere on the body	Approved, Investigational	^326^^,^^327^
Epirubicin	Breast	Low blood counts, Mouth sores, Hair loss on the scalp or elsewhere on the body, Nausea and vomiting, Fatigue, Amenorrhea	Approved	^321^^,^^328^
Etoposide	Testicle, lung, blood, brain, ovarian	Low white blood cell count, Low platelet, Hair loss, Menopause, Loss of fertility, Nausea and vomiting	Approved	^122^
Idarubicin	Blood	Pain along the site where the medication was given, Low blood counts, Urine may appear red, red-brown, orange or pink from the color of the medication for one to two days after you receive a dose, Nausea or vomiting, Mouth sores (in the first week after therapy), Hair loss on the scalp or elsewhere on the body, Diarrhea/abdominal cramps	Approved	^329^
Ifosfamide	Testicle, blood, bladder, bone, pancreas, cervix, breast, stomach, breast, endometrium, lung, ovary	Low white blood cell count, Low platelet count, Hair loss, Nausea and vomiting, Poor appetite, Blood in the urine	Approved	^330^
Irinotecan	Colon, lung	Diarrhea; two types early and late forms, Early diarrhea accompanied by symptoms runny nose, increased salivation, watery eyes, sweating, Fever, flushing, abdominal cramping, Late diarrhea, Nausea and vomiting, Weakness, Low white blood cell count, Low red blood cell count, Hair loss, Poor appetite, Weight loss	Approved, Investigational	^331^
Lomustine	Brain, blood	Low blood counts (bone marrow suppression), Nausea and vomiting	Approved, Investigational	^332^
Mechlorethamine or Chlormethine	Blood, lung, brain	Low blood counts, Nausea and vomiting, Hair loss, Mouth sores, Redness, dryness, irritation with topical use, Loss of fertility	Approved, Investigational	^15^
Melphalan	Blood, ovary, breast, skin	Low blood counts, Nausea and vomiting	Approved	^333^
Mitomycin C	Stomach, intestine, breast, lung, bladder, pancreas, crevice	Low blood counts, Mouth sores, Poor appetite, Fatigue	Approved	^137^
Mitoxantrone	Prostate, blood, breast	Low blood counts, Nausea and vomiting, Fever, increases in blood tests measuring liver function	Approved, Investigational	^334^
Oxaliplatin	Intestine, pancreas, stomach	Peripheral neuropathy, Nausea and vomiting, Diarrhea, Mouth sores, Low blood counts, Fatigue, Loss of appetite	Approved, Investigational	^335^^,^^336^
Temozolomide	Brain, skin	Nausea and vomiting, Constipation, Headache, Fatigue	Approved, Investigational	^337^
Topotecan	Ovary, lung, cervice	Low blood counts, Nausea and vomiting, Hair loss, Diarrhea	Approved, Investigational	338, 339, 340
Thiotepa	Breast, ovary, thyroid, bladder	Low blood counts, Hair loss	Approved, Investigational	^341^
Trabectedin	Ovary	Anemia, Increased liver enzymes, Low white blood cell count, Nausea, Low platelets, Vomiting, Fatigue, Low potassium, Low phosphorous, Decreased appetite, Constipation, Increased CPK	Approved, Investigational	^342^^,^^290^
Purine antimetabolites (purine antagonists)				
Azathioprine	Blood	Black, tarry stools, Bleeding gums, Blood in the urine or stools, Chest pain, Chills, Cough, Fever, Hoarseness, Lower back or side pain, Painful or difficult urination, Pinpoint red spots on the skin, Sore throat, Sores, ulcers, or white spots on the lips or in the mouth, Swollen glands, unusual bleeding or bruising, unusual tiredness or weakness	Approved	^291^
Cladribine	Blood	Fever, Fatigue, Low white blood cell count, Low red blood cell count, Neutropenic fever, Myelosuppression	Approved, Investigational	^343^
Clofarabine	Blood	Leukopenia, Anemia, Infection, Limb pain, Lymphopenia, Low platelet count, Elevated liver enzymes, Vomiting, Nausea, Itching, Neutropenia, Diarrhea, Neutropenic fever, Creatinine increased, Bilirubin increased, Chills, Headache, Fever, Rash, High heart rate, Abdominal pain, Fatigue, Decreased appetite	Approved, Investigational	^293^^,^^344^
Fludarabine	Blood	Low blood counts, Fever, Infection, Weakness, Cough, Nausea and vomiting, Poor appetite	Approved	^294^
Gemcitabine	Breast, ovary, lung, pancreas, bladder	Flu-like symptoms (muscle pain, fever, headache, chills, fatigue), Fever (within 6–12 h of first dose), Fatigue, Nausea (mild), Vomiting, Poor appetite, Skin rash, Low blood counts, Temporary increases in liver enzymes, Blood or protein in the urine	Approved, Investigational	^295^
Mercaptopurine	Blood, intestine	Low blood counts, Liver toxicity, Increased bilirubin, Increased liver enzymes, jaundice, abdominal swelling (ascites)	Approved	^345^
Nelarabine	Blood	Low blood counts, also known as bone marrow suppression (anemia, Fatigue, neutropenia, thrombocytopenia), Nausea	Approved, Investigational	^297^
Pentostatin	Blood	Cough or hoarseness, Fever or chills, Lower back or side pain, Painful or difficult urination, Skin rash or itching (sudden), Unusual tiredness or weakness	Approved, Investigational	^346^
Thioguanine	Blood	Low blood counts	Approved	^347^
Pyrimidine antimetabolites (pyrimidine antagonists)				
Capecitabine	Breast, stomach, intestine	Low white blood cell count, Low red blood cell count, Hand-foot syndrome, Diarrhea, Elevated liver enzymes, Fatigue, Nausea and vomiting, Rash and itching, Abdominal pain	Approved, Investigational	^99^
Cytarabine	Blood	Headache, Low blood counts, nausea and vomiting, Mouth sores, increases in blood tests measuring liver function	Approved, Investigational	^97^
Gemcitabine	Breast, ovarian, lung, pancreas, bladder	Flu-like, Fever, Fatigue, Nausea, Vomiting, Poor appetite, Skin rash, Low blood counts, Temporary increases in liver enzymes, Blood or protein in the urine	Approved	^348^^,^^349^
Fluorouracil	Breast, colon, pancreas, skin, stomach, rectal	Diarrhea, Nausea and possible occasional vomiting, Mouth sores, Poor appetite, Watery eyes, sensitivity to light, Taste changes, metallic taste in mouth during infusion, Low blood counts, white and red blood cells and platelets may temporarily decrease, Skin reactions, Hair thinning, Hand -foot syndrome	Approved	^98^
Raltitrexed	Colon, mesothelium	Increased risk of infection, Breathlessness and looking pale, Tiredness and weakness during and after treatment, Diarrhoea or constipation, Feeling or being sick, Skin problems, Mouth sores and ulcers, Tummy (abdominal) pain, Liver changes, Loss of appetite and weight loss, Dehydration	Approved, Investigational	^102^
Tegafur	Stomach	Myelosuppression, Central neurotoxicity, Gastrointestinal toxicity	Approved, Investigational	^350^
Other antineoplastic agents (belong to one or multiple drug classes)				
Arsenic trioxide (Trisenox®)	Blood	Nausea and vomiting, Cough, Fatigue, Fever, Headache, Rapid heartbeat, Sore throat, Abdominal pain, Diarrhea, Blood test abnormalities (low potassium and magnesium), Shortness of breath, Blurred vision and eye irritation, elevated blood sugar level, swelling of the face, hands, feet or legs, Difficulty sleeping, Rash, Heart rhythm changes, Joint pain, Itching, Numbness or tingling of hands or feet	Approved	^351^^,^^352^
Hydroxyurea	Skin, blood, ovary, breast	Low blood counts	Approved	^301^
Paclitaxel (Taxol®)	Bone, lung, ovary, breast	Low blood counts, Hair loss, Arthralgias and myalgias, pain in the joints and muscles, Peripheral neuropathy, Nausea and vomiting, Diarrhea, Mouth sores, Hypersensitivity reaction	Approved, Vet Approved	^353^
Docetaxel (Taxotere®)	Breast, lung, prostate, stomach	Low white blood cell count, Low red blood cell count, Fluid retention with weight gain, swelling of the ankles or abdominal area, Peripheral neuropathy, Nausea, Diarrhea, Mouth sores, Hair loss, Fatigue and weakness, Infection, Nail changes	Approved, Investigational	^296^
Ixabepilone (Ixempra®)	Breast, skin, lung, blood, prostate, kidney	Peripheral neuropathy, Weakness, Muscle and joint pains, Hair loss, Nausea and Vomiting, Low white blood cell count	Approved, Investigational	^354^
Methotrexate	Blood, breast chromium	Dizziness, Drowsiness, Headache, Hair Loss, Swollen, Tender Gums Decreased Appetite, Reddened Eyes	Approved	^355^
Pemetrexed (Alimta®)	Pleura, lung	Fatigue, Nausea	Approved, Investigational	^305^
Streptozocin or Streptozotocin	Pancreas	Nausea and vomiting, Nephrotoxicity	Approved, Investigational	^356^
Vinblastine	Breast, blood, testicle, bone	Low blood counts, Injection site reactions, Fatigue and weakness	Approved	^307^
Vincristine	Blood	Hair loss, neurotoxicity, alopecia, myelosuppression	Approved, Investigational	^357^
Vindesine or Eldisine	Blood	Myelosuppression, Neurotoxicity	Approved, Investigational	^358^
Vinorelbine	Lung, blood, ovary, breast	Low blood counts, Nausea or vomiting, Muscle weakness, Constipation	Approved, Investigational	^359^^,^^360^

## Etoposide

Etoposide is a semi-synthetic derivative of a plant glycoside called podophyllotoxin.^117^^,^^118^ It causes DNA damage by altering DNA topoisomerase-II function, and posing G2 cell cycle arrest.^119^ Etoposide creates a ternary complex with DNA and the topoisomerase–II complex, preventing the latter's binding to the DNA strands, while in doing so, it causes damage to DNA strands.^120^ Cancer cells depend on this enzyme more than healthy cells, as they divide more quickly; therefore, it causes defects in DNA synthesis that promotes apoptosis of the cancer cells.^121^ Etoposide is effective against several aggressive malignancies including Kaposi's sarcoma, Ewing's sarcoma, testicular disease, lung disease, lymphoma, non-lymphocytic leukemia, and glioblastoma multiforme (Table 1).^122^

## Camptothecin and its derivatives

Camptothecin is a long-known topoisomerase inhibitory compound of natural origin. It was initially extracted from the bark of the tree *Camptotheca acuminata*.^123^^,^^124^ Camptothecin is a cytotoxic alkaloid comprised of a pentacyclic ring structure containing a pyrrole (3,4-β) quinoline moiety, an S-configured alpha-hydroxy lactone form, and a carboxylate form.^125^ In later years, many more effective derivatives of this topoisomerase inhibitor have been synthesized and are effectively being applied in cancer chemotherapy.^126^ The most recognized drug of this class, topotecan, is used against the advanced cancers of the ovary and lung. It targets topoisomerase-I, an enzyme responsible for easing the torsional strain in DNA by inducing single-strand breaks during replication.^120^ The inhibition of topoisomerase-I by the active compounds may lead to sudden inhibition of DNA replication and transcription, causing growth arrest (Table 1).^127^ In the physiological conditions, topotecan remains in equilibrium with its carboxylate structure.^128^ Its active lactone can intercalate between DNA bases in the topoisomerase-I cleavage complex.^129^ The coupling site of topotecan in the cleavage complex interferes with the topoisomerase-I while reiterating the scratched DNA strand in the wake of releasing the strain. Such intercalation traps the topoisomerase-I in the cleavage complex bound to the DNA.^130^ Another well-known drug of this class is irinotecan that has shown efficacy against late-stage colorectal carcinoma, aggressive growths in cancers of the lung, cervix, ovary, colon, etc.^131^^,^^132^

## Actinomycin D (dactinomycin)

A transcription inhibitor chromopeptide molecule actinomycin D (also called dactinomycin) binds to DNA while initiating transcription at initiation complex and inhibits RNA elongation steps.^133^ It can intercalate, preferably between guanidine and cytosine, or bind the DNA strand's minor grooves to form a highly stable drug-DNA complex, preventing DNA unwinding and thus stalling the progress of the RNA chain.^134^ Dactinomycin was initially obtained from *Streptomyces* sp., identified as an antibiotic agent, but later its anti-tumor potential was recognized, and thus it was inducted in the chemotherapy as an anticancer antibiotic to treat Ewing's sarcoma, Wilms' tumor, rhabdomyosarcoma, trophoblastic neoplasm, testicular and ovarian cancers, etc (Table 1).^135^

## Mitomycin C

Mitomycins, a class of natural quinones, are also derived from *Streptomyces caespitosus* and are known for their transformation and competence, enhancing potential in bacterial cells. It is also a known antineoplastic agent as it slows down fibroblast cell growth in glaucoma.^136^^,^^137^ These bioreductive alkylating agents (especially mitomycin C derivatives) are also utilized as anti-cancer therapeutics because their structural modification impedes the cell cycle during G1 and S phase transitions. Its reduction to form mitosene can lead to the N-alkylation of DNA bases.^138^ It can lead to inhibition of replication by cross-linking at adenine N6 and guanine O6 and N2.^139^ It is effectively used in the palliative treatment of non-small cell lung carcinoma and other solid tumors of breast, colorectal, oesophageal, pancreatic, and cervical origins. Porfiromycin is an N-methyl derivative of mitomycin C that is found effective against cancer of head and neck regions (Table 1).^140^

## Daunorubicin (rubidomycin)

Daunorubicin causes a break in DNA strands by actuating topoisomerase-II and producing quinone-type free radicals. Daunorubicin, otherwise called daunomycin, is a chemotherapy medicine used to treat cancer growth in acute myeloid leukemia (AML), acute lymphocytic leukemia (ALL), chronic myelogenous leukemia (CML), and Kaposi's sarcoma, etc.^141^^,^^142^ It is applied by infusion into a vein, and following its intercalation with DNA, it hinders macromolecular biosynthesis. This predominantly obstructs the movement of the enzyme topoisomerase-II, which loosens up the DNA supercoiling.^143^ Daunorubicin impedes the topoisomerase–II complex after it unties the DNA chain for replication and thereby it represses the DNA double helix from resealing and therefore interferes with the replication to proceed (Table 1).^144^^,^^145^

## Bleomycin

Another *Streptomyces* compound with anti-tumor activity bleomycin is mostly used against Hodgkin's lymphoma, ovarian and testicular cancers. It chelates copper or iron, produces superoxide radicals, and intercalates between DNA strands – causes chain scission.^146^ Emerging evidence proposes that bleomycin also hinders the consolidation of thymidine into DNA strands, and the cleavage of DNA relies upon oxygen and metal particles *in vitro*.^147^ The specific mechanism of DNA strand scission is uncertain, yet it has been proposed that bleomycin chelates metal particles (principally iron), delivering a pseudoenzyme that responds with oxygen to create superoxide and hydroxide free radicals that divide DNA; however, little myelosuppression is the unique highlight.^148^ The drug has also been effective against squamous cell carcinoma of the skin, and cancers of the mouth, head, neck, genitourinary tract, and throat (Table 1).

## Lateral damage: Putting multiple organs at risk

Aggressive tumors are primarily marked with uncontrolled and extensive cell division that may compromise the normal physiology of other non-transformed cells in the vicinity of affected organs/tissues. In many ways, cancer cells achieve super-specialized metabolic and survival potential with the capacity to tolerate extreme stresses like low oxygen and nutrient supplies. Cancers might start from mutation of one type or the other, but as the disease progresses,^149^^,^^150^ multiple mutations and genetic abnormalities accumulate that lead to increased tumor heterogeneity.^87^ The heterogeneous nature of tumor mass plays a vital role in developing resistance against most of the drugs that we have described here. This leads to a challenge of its kind, leaving doctors with the only option of changing the medication after certain intervals as one type of drug stops acting against tumors. In many cases, the combination therapy that includes more than one drug at a time could be an effective strategy against some form/type of cancers, such as cancers affecting bone marrow (leukemia) and lymph nodes (lymphoma). Clinical trials help a lot in understanding the effects of new combinations and thus have rapidly improved the cancer chemotherapy paradigm.

The sustainability of chemotherapy relies upon its continuous effect on cell division and tumor growth. As discussed above, the majority of the treatments work by compromising the stability or integrity of nucleic acids that tend to stall the cell cycle or activate programmed cell death pathways. Other drugs may act by generating extreme proteotoxic stress inside the cells by interfering with the protein folding or degradation machinery, including chaperone, proteasome, and autophagy.^151^, ^152^, ^153^, ^154^ The idea is to generate cellular stresses so that the cancer cells that are already under compromised physiology may die while other cells can survive. However, it is evident from the above descriptions that most of these drugs act by interfering with one or multiple cellular pathways of extreme importance.^155^ Therefore, chemotherapeutic drugs are mostly referred to as cytotoxic agents, causing a plethora of side effects. In most cases, long-term treatments may lead to enormous toxicity causing severe damage or failure of multiple organs. This may lead to enormous physiological and psychological stress to the patients under chemotherapeutic medication. Table 2 summarizes the adverse effects of various chemotherapeutic agents and the current stage of their clinical progress. The side effects of these drugs may depend on many factors and variables, such as type, stage, and location of the tumor, along with age, health, and other clinical aspects of the patients. The most common and readily visible side effects originate from the gut that may lead to nausea, vomiting, constipation, and diarrhea.^156^^,^^157^ Most of the anticancer drugs can directly interrupt the cell division pathways; therefore, the first line of affected organs may also include those which are formed with continuously dividing cells, such as skin, hair, and intestinal linings.^158^^,^^148^ Skin rashes, alopecia, and mucositis are the most visible side effects that one can observe in patients undergoing chemotherapy.^157^^,^^159^ Radiation also contributes significantly to these debilitating changes. A general problem observed in many patients is fatigue that can occur due to multiple factors, including depression, anemia, high doses of radiation therapy, etc.^160^

Chemotherapeutic drugs may also directly injure the bone marrow and cause a steep decline in blood cell count, exposing patients to several other types of possible infections.^161^ Thrombocytopenia, a condition of excessive bleeding because of lower platelet numbers, is observed more frequently in patients taking alkylating and/or antimetabolites agents.^162^ Alkylating agents may further trigger conditions, such as hemorrhagic cystitis leading to microscopic haematuria, nocturia, dysuria, etc. along with mild or intense suprapubic pain.^163^ Whereas, antimetabolites can generate enormous toxicity in vital organs, like the liver and kidneys.^164^^,^^165^ The most devastating of all is their direct impact on cardiovascular health. Different classes of chemotherapeutic drugs have varying impacts on cardiac cells, leading to multiple types of systemic conditions adversely affecting patients' survival.^166^ Loss of libido and sexual dysfunctions are other conditions caused due to long-term exposure to these drugs, which affects not only physical well-being but also the patient's psychological health (Table 2).^167^^,^^168^

These drugs and radiation therapies may have several direct or indirect impacts on cognitive functioning leading to a condition, which is now commonly referred to as chemo brain.^169^^,^^170^ This is a condition of the inability of someone undergoing chemo-drug therapies to do their routine cognitive functions, which may be related to their memory, cognition, and behavior.^171^ The drugs may adversely impact communication, reasoning, learning, problem-solving, memory storage, concentration, following commands, etc. Damage of optic and the ocular motor nerves is also observed in several patients taking antineoplastic drugs that may lead to the weakening of vision as well.^172^^,^^173^ The majority of chemotherapeutic agents affect these tissues in a dose-dependent manner; though, there are differences in their sensitivity to individuals due to enormous factors such as metabolism efficiency, genetic makeup, other medication, and environmental factors (Table 2).^174^ Despite enormous toxicities and side effects prevalent in the public domain, the chemotherapy medicines remained the most immediate and viable therapeutic option in most cases, where we can see the cancer cells but cannot remove them through surgery or source of radiation. To address various above-described detrimental physical and psychological changes, modern-day clinical practices involve a comprehensive team of doctors and specialists for the patients who are undergoing long-term treatments and are more prone to develop severe physiological complications.

## Recent advancements and next-generation therapeutic approaches

**Figure 3:**
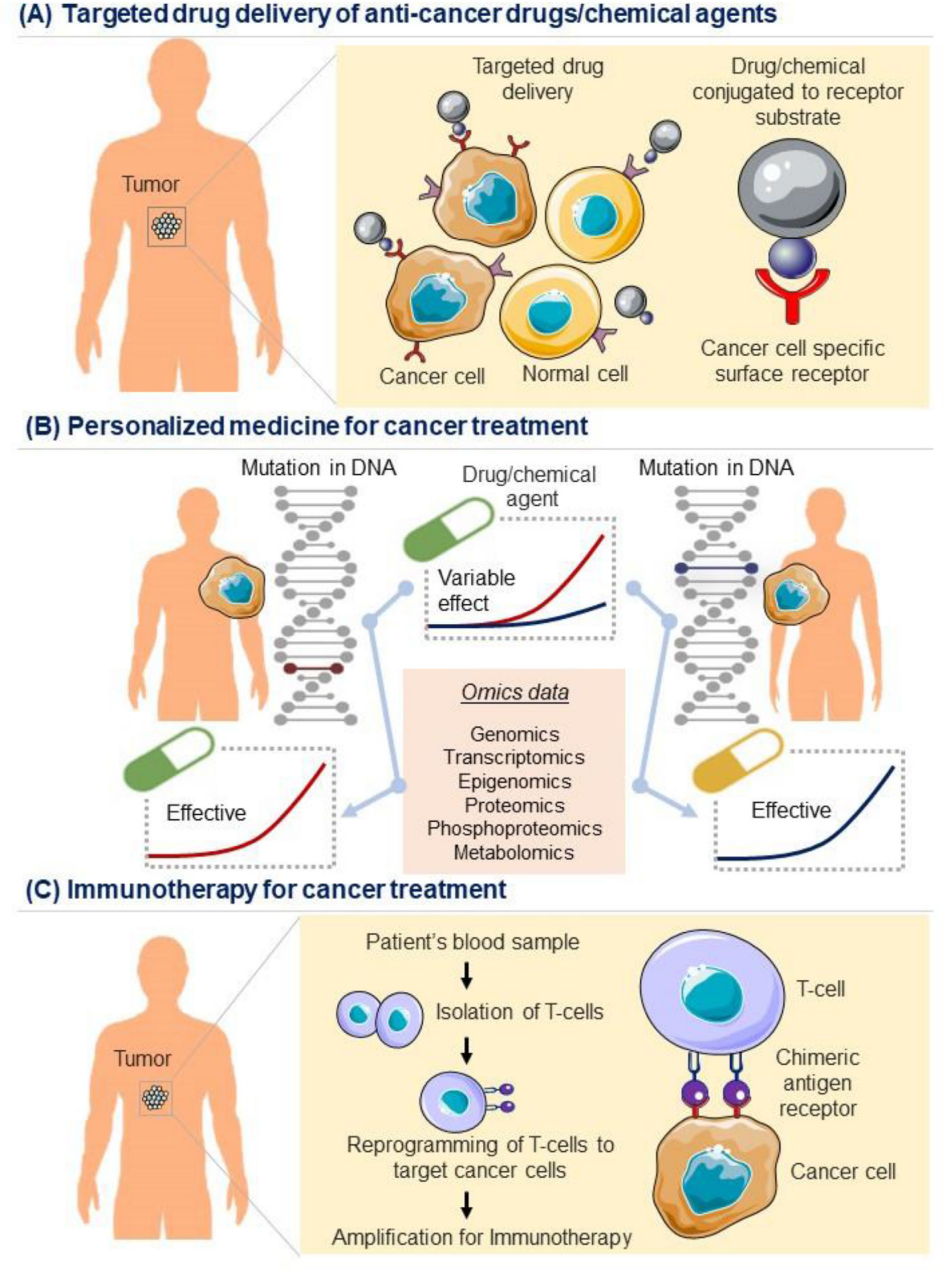
Schematic diagram showing targeted cancer therapeutic approaches. **(A)** Model showing targeted drug delivery approach of anti-cancer drugs/chemical agents conjugated with receptor substrate specific to the cancer cell receptor. **(B)** Model showing the building of a personalized medicine approach based on omics data and drug response study targeting specific mutation/signature in individual patients for precision cancer therapeutics. **(C)** Model showing immunotherapeutic regime involving reprogramming of T-cells to target cancer cells by a chimeric antigen receptor for their application in cancer immunotherapy.

Since the early 19th century, rigorous investigations and clinical studies were carried out around the world. Several research groups have made landmark discoveries during and after the Second World War. Following the development of a fundamental description of the disease and associated pathways, the focus shifted majorly towards advancing the diagnosis and treatment methods of the disease. Despite enormous progress being made in the second half of the last century, not much could have been achieved in successfully curing most of the cancers. One primary reason behind the failures is our inability to diagnose the oncogenic changes inside the body at the early stages of cancer development. In recent years, the concept of liquid biopsies has emerged that primarily relies on the screening of mutated DNAs, proteins, RNAs, and other genetic markers in the patient's blood samples.^175^, ^176^, ^177^ This practice can help diagnose the disease at a much earlier stage than we do now, using the traditional tissue biopsies and imaging methods.^178^ New technological tools, like advanced software and robotic platforms, have also speeded cancer diagnosis and treatment. In this subsequent section taking chemotherapy into account, we compressively reviewed the recent progress made in the targeted drug delivery, personalized medication, and immunotherapy (Fig. 3).

## Targeted drug delivery

The major drawback of most chemotherapeutic treatment plans is their non-specificity or inability to ascertain and target cancerous cells directly. Therefore, this lack of specificity poses a major therapeutic limit reflected in the failure of delivering drugs locally to the cancer tissue mass. In the past few decades, many new approaches have been constituted and tested, and have given hopes for future drug delivery (Fig. 3A). Nanoparticle-based delivery methods, targeted antibodies, aptamer functionalization, and specific drugs like Herceptin (in breast cancer) offered the promise that can harness great success in the upcoming years.^179^, ^180^, ^181^ Cancer cell-directed antibodies are at the forefront of targeted drug delivery methods and have accelerated the hunt for more effective or potentially improvised approaches.^182^^,^^183^ Different types of nanocarriers and formulations of nano-drugs have also improved the delivery of drug compounds to the cancer cells.^184^, ^185^, ^186^ The most significant advantage of targeted drug deliveries is that the enormous drug-mediated toxicities on other adjacent tissues and organs can be minimized. We can also avoid most of the collateral damage leading to stress and organ failures. However, the effectiveness of many of these delivery methods in patients is still under clinical evaluation. Further details regarding these delivery systems can be accessed in the previously published reports.^187^^,^^188^

## Personalized medication

Given the heterogeneity of tumor cells, the cancer of one patient is different from that of another in terms of accumulated genetic abnormalities, its pace and progression, the response to the treatment, and the chances of developing resistance to the administered drugs.^45^ The current technologies have made it possible to get insights into this disease heterogeneity or explore molecular variations at the individual level, thereby tailoring the treatment regime as per the personalized needs of every cancer patient.^149^^,^^189^ This helps in choosing the most appropriate drugs and their dosages for a better outcome in cancer management.^190^ For instance, dabrafenib and vemurafenib are directed against *BRAF* gene mutation, while trastuzumab works well against HER-2 mutated cells.^47^^,^^191^^,^^192^ The idea here is to dive deep into the omics (genomic, transcriptomic, proteomic, metabolomic, etc.) data of the patient to identify the major molecular players fostering the transformed cells, and design the treatments against these clinically actionable targets (Fig. 3B).^155^^,^^193^^,^^194^ Different types of mutations have been observed to be frequent in specific subtypes of cancer. Therefore, measuring and manipulating these driver mutations based on an individual patient is expected to yield better results. Following are some examples of drugs that are potentially effective in patients harbouring some specific type of genomic alteration. A mutation in anaplastic lymphoma kinase (ALK) was reported to drive tumor formation in 5% of the non-small-cell lung cancer population and led to the discovery of ALK blockers such as crizotinib and ceritinib.^195^ An oral BRAF inhibitor, namely vemurafenib has been shown to possess a greater affinity for the mutant BRAF^V600E^ than the wildtype BRAF, and therefore was recommended to the patients carrying the above mutation in the tumors.^196^ Several potential targets for novel drugs have been identified using high-throughput screening technologies, and various compounds against them have been recently approved or are under investigation. Similarly, alterations in phosphoinositide 3-kinases (PI3K)/protein kinase B (AKT)/mTOR pathway have also been frequently found in many solid tumors. Among these, activation of phosphatidylinositol-4,5-bisphosphate 3-kinase catalytic subunit alpha (PIK3CA) and loss of phosphatase and tensin homolog (PTEN) mutations are the most common. Basket trials have been conducted to evaluate the effectiveness of adding alpelisib, a specific p110α PIK3CA inhibitor to the hormonal treatment for PIK3CA mutant luminal breast cancer patients. The results emphasize the importance of checking for this molecular alteration in patients and following the tested treatment regime for better anti-tumor response.^193^ Next, aberrant changes in the fibroblast growth factors (FGF) signaling pathway have also been associated with the initiation and progression of oncogenic events. Different types of genetic alterations in FGF receptor amplification, mutations, and gene-fusion have been shown to respond variably to certain drugs, and hence demand molecular profiling for the selection of effective drugs.^46^^,^^197^ Another promising example is of using poly ADP ribose polymerase (PARP) inhibitor olaparib for the treatment of breast cancer susceptibility gene (*BRCA*)-mutant cancers for better targeting of cancer cells, without much effect on the normal cells.^198^^,^^199^ On the cytoprotective side, recently a natural alkylated withanolide *viz.* 2,3-Dihydro-3beta-methoxy withaferin-A was shown to protect normal cells against a variety of stresses enrolled in cancer therapeutics.^200^ The idea of precision or personalized medicine is new but has been beneficial for lots of patients; however, the cost of these new generation approaches for genome profiling is still high, making it unaffordable for a large population of the world.^201^

## Antimicrobial peptides

**Figure 4:**
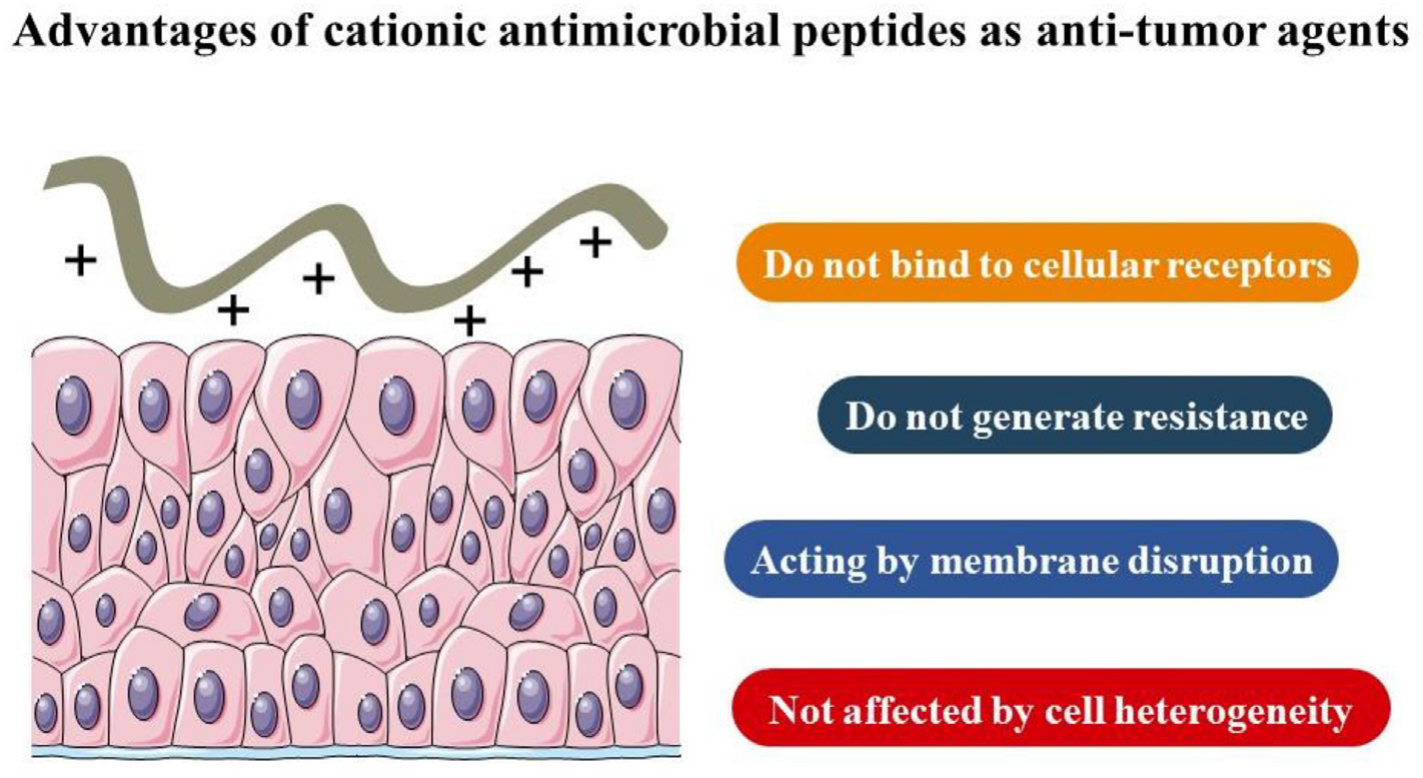
Advantages of antimicrobial peptides as anti-tumor agents.

Advances in cancer therapy have prompted scientists to look for natural tumoricidal chemicals, such as antimicrobial peptides (AMPs), that could be used for cancer treatment.^202^, ^203^, ^204^ In addition to antibacterial action, several AMPs have been shown to have intrinsic anticancer potential.^205^^,^^206^ These molecules constitute an intriguing new resource for anticancer drugs, and they provide several distinct therapeutic advantages over other anticancer treatments since they do not bind directly to any particular receptor in cancer cells.^207^ The polycationic and amphipathic character of AMPs has been postulated as a possible explanation for the preferential interaction of AMPs with cancer cells, as compared to normal cells.^208^^,^^209^ Membrane disruption has been identified as a critical stage in the microbial killing mechanism of several AMPs. These anticancer effects of AMPs are unaffected by molecular heterogeneity inside a tumor or across tumors, nor are they counteracted by cancer cells' flexibility and alternate survival pathways, as is commonly found in cancer resistance (Fig. 4).^204^^,^^209^^,^^210^

Cathelicidins are members of the antimicrobial peptide family found in granules of vertebrate neutrophils.^211^ The only human cathelicidin is the cationic peptide LL-37. Cathelicidins are composed of a highly conserved N-terminal signal peptide including a cathelin domain and a C-terminal cationic antimicrobial peptide. Apart from their antimicrobial activity, cathelicidin-derived antimicrobial peptides exhibit a range of biological roles, including antiviral, antifungal, anticancer, and immunomodulatory properties.^212^^,^^213^ LL-37 exhibits anticancer activity against oral cancer cells and has been identified as a molecular target for colon cancer.^214^ Immunohistochemical staining of colon cancer tissue showed that LL-37 is abundantly expressed in normal colon mucosa but downregulated in colon cancer tissues due to DNA methylation in the LL-37 promoter.^215^

In human oral squamous cell carcinoma SAS-H1 cells, the active domain of LL-37 induces mitochondrial depolarization, which results in caspase-independent apoptosis. Furthermore, mice lacking cathelicidin are more susceptible to azoxymethane-induced colon carcinogenesis.^216^

Many important advancements have been achieved using antimicrobial peptides against colorectal cancer.^217^ Using LL-37 on the surface of magnetic nanoparticles led to a significant reduction in cell viability of colon cancer cell lines, inducing apoptosis at a much higher rate than in the case of LL-37 peptide alone.^218^

Fan et al created a biodegradable and injectable thermosensitive hydrogel containing docetaxel and LL-37 polymeric nanoparticles. Intraperitoneal injection of this hydrogel substantially inhibited the development of HCT116 carcinomatosis in a mouse model of colorectal carcinoma.^219^

In comparison to existing chemotherapeutic agents, the flexible nature of peptide structures, along with non-receptor activity, might confer strategic benefits on AMPs for the treatment of colon cancer in the future.^220^ New treatment approaches for patients with advanced-stage, unresectable melanomas continue to be of significant clinical interest, as these patients cannot be treated surgically and have only a modest or temporary response to chemotherapy and radiation. In phase 1 clinical trial NCT02225366, intra-tumoral injections of LL-37 are being explored for melanoma patients with cutaneous metastases to determine the most effective dose of LL-37 that could be administered. Patients will receive weekly intra-tumoral injections of LL-37 (starting dose, 250 μg/tumor) every 7 days for up to 8 weeks. Researchers are also interested in understanding whether LL37 can activate the immune system, which could help in the control of the disease.^221^

Although AMPs have the potential to develop into a more effective and safer alternative to conventional cancer therapy, future research and development of anticancer peptide therapeutics will most likely focus on the poor pharmacokinetics, the control of selectivity, toxicity, and routes of administration through the establishment of appropriate *in vivo* models.^222^

## Immunotherapy

Supplementing our immune system by devising immunotherapy strategies is another highly-specific and largely promising approach to address the problem of late-stage cancer therapeutics.^223^^,^^224^ Several new approaches have been developed in the last decade to modify and train a patient's immune system to search, identify and target the transformed cells inside the body by recognizing multiple cell surface markers.^225^ For instance, supplementing the treatment regime with immunotherapy has significantly improved the success rate of the chemotherapeutic drugs in many cancer types, especially the triple-negative breast cancers.^226^, ^227^, ^228^ The immunotherapy drugs are given alone or in combination with chemotherapy drugs to achieve better clinical outcomes.^229^ Prophylactic and therapeutic vaccines have been developed to help patients' immune systems towards learning to tackle the transformed cells.^230^ Atezolizumab is an example of an anti-cancer immune drug that helps to dissolve hard-to-treat breast cancers.^231^, ^232^, ^233^ Tisagenlecleucel, an FDA-approved drug, treats B-cell lymphoblastic leukemia by effectively engineering the chimeric T cell receptor (CAR-T) of the patient (Fig. 3C).^234^ Some treatment plans also include interferons and interleukins to jump-start the fight against cancer cells by boosting the patient's immune system. Moreover, the field of immunotherapy is gradually evolving and is beyond the scope of our present article.

## Combination therapy

Combination therapy may offer the advantages of the fast tumor regression of targeted therapies with the long-lasting responses induced by immunotherapy.^235^ Immunotherapy may be able to consolidate the dramatic tumor responses achieved with targeted therapy into durable, long-lasting remissions, in which sustained host responses targeting multiple antigens may reduce the risk of developing potentially lethal drug-resistant clones.^236^ Combining cancer vaccines with immune checkpoint inhibitors (ICIs) such as those targeting cytotoxic T lymphocyte-associated protein 4 (CTLA-4) and programmed cell death protein 1/programmed cell death ligand 1 (PD-1/PD-L1) is one approach to avoid T cell anergy.^237^^,^^238^ Moreover, T-cell and NK-cell invasion may be facilitated by targeted drugs that modify the tumor endothelium.^239^ Gemcitabine, for example, is a deoxycytidine analog that induces tumor cell death, which results in antigen cross-presentation and cross-priming.^240^^,^^241^ Carboplatin, pemetrexed, and pembrolizumab are now commonly used in the treatment of non-small cell lung cancer as first-line agents.^242^ For triple-negative breast cancer, nab-paclitaxel and atezolizumab (anti-PD-L1) were recently authorized based on an improvement in progression-free survival (PFS) over chemotherapy.^243^

Adenosine receptor antagonists (ARs) are involved in regulating tumor progression.^244^ The four ARs discovered so far, A_1_, A_2A_, A_2B_ and A_3_, belong to the class A family of G protein-coupled receptors (GPCRs).^245^ The A_2B_R seems more promising as a target in chemotherapeutic interventions,^246^ whereas the A_2A_R is the main target in the immunotherapeutic approach.^247^^,^^248^ The existence of dual compounds, i.e., molecules acting on the A_2A_R and on the A_2B_R, has prompted their use in both immunotherapeutic and chemotherapeutic interventions.^249^^,^^250^

Epigenetic modulators may have a dual function in the tumor microenvironment, boosting both antigen expression and T cell effector activity.^251^^,^^252^ ICI-induced reactivation of exhausted T cells is linked with considerable chromatin remodeling, indicating that epigenetic modification may relieve certain patients with exhausted T cell function.^253^^,^^254^

While many strategies focus on modifying the native tumor microenvironment, an alternative strategy involves the direct infusion of engineered immune cells or cellular receptors into the patient via adoptive cellular therapy with the goal of increasing the number of effector cells that recognize tumor-expressed antigens.^255^ Upon reinfusion, lymphocytes grown *ex vivo* may respond more potently to tumor antigen since they have not been continuously exposed to tolerogenic microenvironmental signals.^256^ Tumor infiltrating lymphocytes (TIL) treatment,^257^ chimeric antigen receptor (CAR) engineered T-cells,^258^^,^^259^ and TCR-engineered cell therapy^260^^,^^261^ are the three main forms of adoptive T cell therapy currently being investigated.^257^^,^^262^^,^^263^

Injecting oncolytic viruses directly into the tumor microenvironment is an alternate technique for improving tumor antigen recognition and strengthening T cell responses.^264^ Talimogene laherparepvec (T-VEC) is a modified herpes simplex virus that is injected intra-lesionally into the melanoma tumor in individuals who have failed to get their tumor removed. It induces immediate lysis of tumor cells and the production of granulocyte-macrophage colony-stimulating factor (GM-CSF), which serves as a cytokine to stimulate the recruitment, maturation, and activity of antigen presenting cells (APCs).^265^

Several studies combining immunotherapy with molecularly targeted treatment, either concurrently or sequentially, have shown hepatotoxicity as a major concern.^266^ Combinations of dabrafenib, trametinib, and anti-PD-1 treatment have been associated with a greater risk of grade 3/4 adverse events in melanoma than would be expected with targeted therapy alone.^256^

Emerging drugs are beginning to take a more comprehensive perspective of the tumor microenvironment (TME) and are revealing complementary techniques to simultaneously restore and enhance immune activity and promote therapeutic synergy.^267^^,^^268^ Future research of combined therapy should continue to concentrate on exploring the intricate interplay between targeted drugs and immunotherapy, as well as optimizing parameters such as administration time, dose, and sequencing that may enhance the therapeutic index.^240^

## Conclusions and future perspectives

Chemotherapy is a largely investigated and clinically assessed approach in cancer therapeutics. In this report, we summarized the present status of chemotherapy and several drugs/agents presently enrolled in the clinical practice to provide an overview of how the field has evolved over the years and has traced the possibilities and druggability of diverse cancers in the clinic. New trends are evolving in the drug discovery paradigm that includes designing new derivatives of old drugs with high efficacy and lower toxicities.^269^^,^^270^ Several natural compounds isolated from microbial, plants, or natural sources were suggested to hold enormous potential to target stress response pathways by mounting a hyperactive response instigating cancer cell death.^271^, ^272^, ^273^, ^274^ Natural compounds/agents as chemotherapeutic drugs demonstrated several advantages over synthetic ones, owing to their less cytotoxicity, cost, and straightforward production/extraction process.^273^^,^^275^^,^^276^ Therefore, recognition and attenuation of treatment toxicity in cancer chemotherapy will be a key factor in determining the success of its regimes in the future.

Revised clinical strategies further underlined that combination therapy could emerge as a promising therapeutic approach to treat diverse cancers in the future. Combination strategies comprise an obvious overlap of the chemotherapeutic regimes with the targeted drug delivery, personalized medicine, and immunotherapy. It may not only improve the efficacy of enrolled treatment but can also elicit the responsiveness of certain tumors towards their effective therapeutic targeting in the clinic. Among these, personalized combination therapies that target individual tumor types based on their molecular signatures may offer a great promise. With acquiring more personalized insights in the chemotherapeutic treatments, a greater emphasis will be on individual regimes that could meet a high clinical success.^277^ Therefore, chemotherapy beyond its conventional clinical efficacy offers to improve the clinical outcome in combination therapies. Translation of these combined approaches in the clinics is yet to see a complete success and thus warrants more clinical investigations.

Conclusively, clinical practice of cancer chemotherapy has achieved considerable success, however, it still holds scope to improve, particularly in terms of efficacy and safety of enrolled chemotherapeutic regimes. Therefore, it further requires concerted efforts and careful investigations assessing the efficacy of combined therapeutic regimes. In summary, more investigative focus on drug design in individual or combined regimes and rigor on their clinical trials involving careful patient selection and stratification are warranted to achieve the greater therapeutic success of chemotherapeutic regimens in the clinic.

## Conflict of interests

The authors declare no conflict of interests.

## Funding

This research was funded by "Agencia Canaria de Investigación, Innovación y Sociedad de la Información (10.13039/501100007757ACIISI) del Gobierno de Canarias” (No. ProID2020010134), and óCaja Canarias (Project No. 2019SP43).
